# In Vivo Imaging of a Photoactivatable Platinum Prodrug by Metal-Centered Radiolabeling

**DOI:** 10.1021/jacs.6c05292

**Published:** 2026-05-30

**Authors:** George Firth, Jana Kim, Huayun Shi, Kavitha Sunassee, Oene Zwaagstra, Karlijn van der Schilden, Philip J. Blower, Peter J. Sadler, Cinzia Imberti

**Affiliations:** † School of Biomedical Engineering and Imaging Sciences, 4616 King’s College London, London SE1 7EH, U.K.; ‡ Department of Chemistry, 2707 University of Warwick, Coventry CV4 7AL, U.K.; § NRG PALLAS, Petten 1755 LE, The Netherlands

## Abstract

The in vivo fate of metal-based photoactivatable prodrugs is often unknown or inferred from structurally modified analogues rather than the drug candidates themselves, which may lack suitable spectroscopic reporters. New methods are needed to reveal authentic biological behavior. We report the multistep radiosynthesis of a photoactivatable platinum­(IV) prodrug and new insights into its biomolecular properties from single-photon emission computed tomography imaging using (metastable) platinum-195m. Metal-centered radiolabeling preserves both the chemical identity and photoreactivity of the non-radioactive prodrug and enables direct radioanalytical tracking in biological media and in vivo. Whole-body imaging in healthy and tumor-bearing mice revealed rapid renal clearance of the drug, with unexpected and persistent accumulation in the bladder wall, a behavior not observed for cisplatin or structurally-modified analogues. These results demonstrate how metal-centered radiolabeling enables direct interrogation of the in vivo fate of metal-based photoactivatable drug candidates and reveals biological behavior information that is inaccessible using indirect labeling strategies.

## Graphical abstract

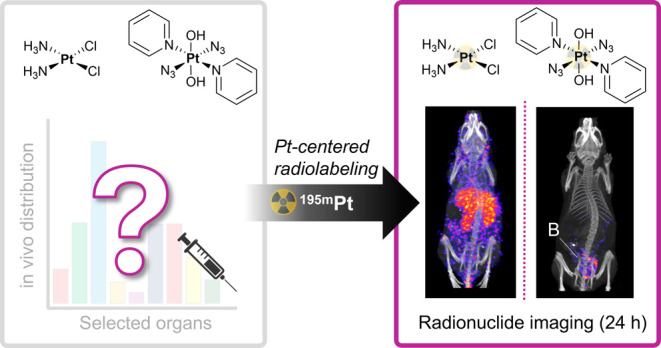

Metal-based photodynamic and photoactivatable prodrugs offer a promising strategy for precision anticancer therapy by enabling spatially- and temporally controlled drug activation with light, with the potential to reduce off-target toxicity and improve therapeutic selectivity.^1–6^ A central challenge in the development of such agents is that therapeutic efficacy depends on aligning light irradiation with drug accumulation and clearance in tumors. The ability to visualize photoactivatable drugs in living systems would enable rational optimization of irradiation protocols and accelerate preclinical translation.

Radiolabeling at the metal center, often the key pharmacophore, offers a unique solution. It enables the intact prodrug and its metal-containing photoproducts to be visualized without structural modification. For platinum-based photoactivatable drugs, the metastable radioisotope ^195m^Pt is particularly well suited for this purpose, combining γ‑emissions compatible with single-photon emission computed tomography (SPECT) with a 4-day half-life enabling longitudinal studies. Historically, the broader application of ^195m^Pt has been limited by challenges in isotope production, including low yields and specific activity, as well as the limited sensitivity and spatial resolution of earlier gamma imaging instrumentation, but recent advances in both areas have renewed interest in its use.^7^ Notably, ^195m^Pt decays to stable ^195^Pt, so that radiolabeling provides a temporarily traceable version of the same platinum drug rather than a permanently modified analogue. Early studies demonstrated the feasibility of this approach by clinical imaging of ^195m^Pt-labeled cisplatin, but further application of platinum-centered radiolabeling has since remained limited to structurally simple coordination motifs.^8–11^ The application of this strategy to photoactivatable prodrugs would provide a major advance in their development. We report a multistep radiosynthesis of the light-activated Pt agent *trans*,*trans*,*trans*-[^195m^Pt­(OH)_2_(N_3_)_2_(pyridine)_2_] ([^195m^Pt]­azoPt, Figure 1A)^12,13^ and determination of its in vivo pharmacokinetics and biodistribution by ^195m^Pt-SPECT imaging.

**Figure 1:**
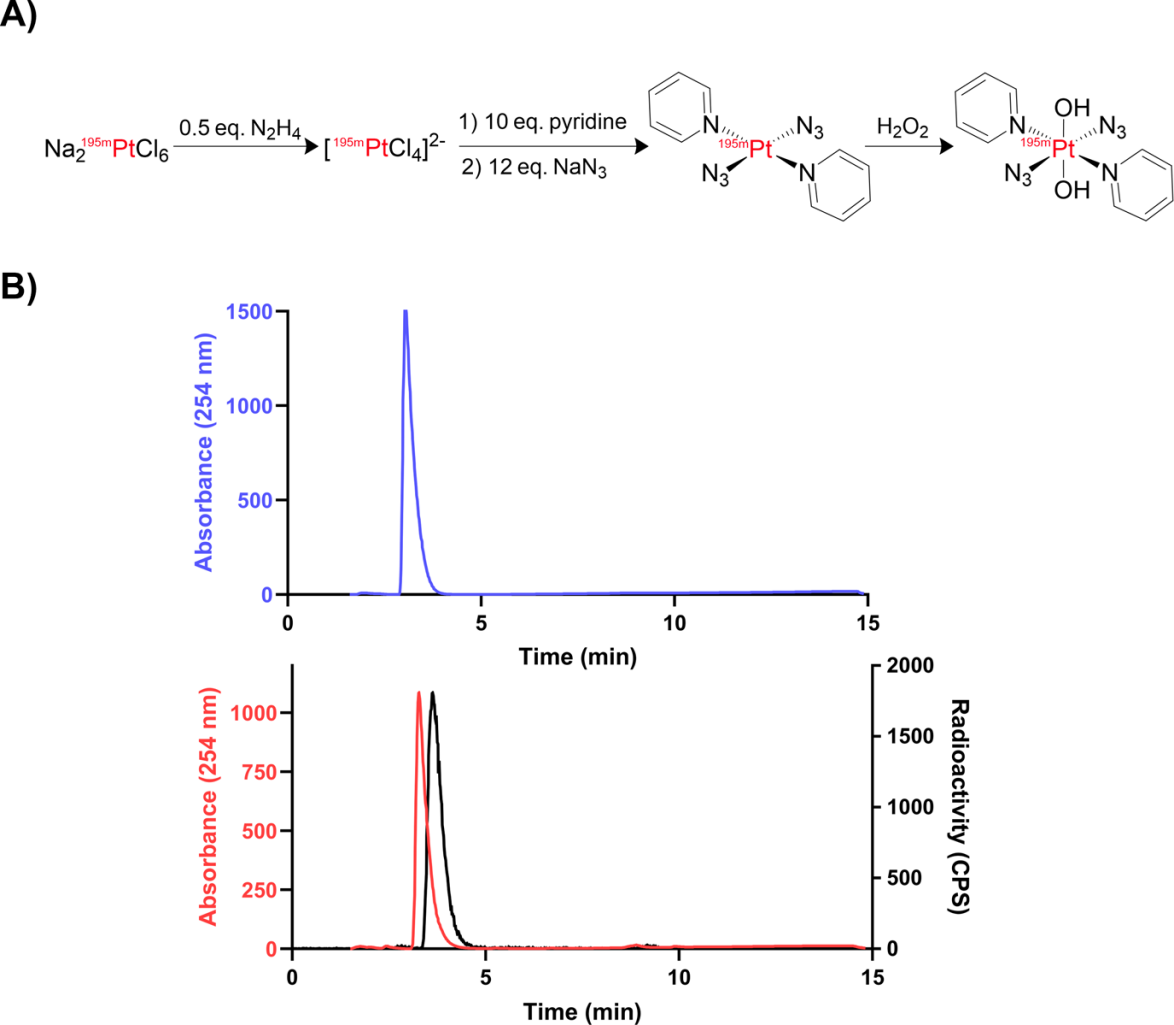
Radiosynthesis of [^195m^Pt]­azoPt. (A) Single-vessel radiosynthetic route enabling preparation of the photoactivatable Pt­(IV) prodrug within 9 h. (B) RP-HPLC chromatograms of non-radioactive azoPt (top) and [^195m^Pt]­azoPt (bottom) showing identical UV traces; the radioactive signal (black) coelutes with the UV peak (red), and the small difference in retention time arises from the in-series configuration of the UV and radioactivity detectors (CPS = counts per second).

This complex serves as a prototype for a broad class of photoactivatable Pt­(IV)-azido complexes with tunable photophysical properties^14,15^ and provides a benchmark for extending ^195m^Pt radiolabeling to investigate the biological behavior of other, non-photoactivatable Pt­(IV) anticancer prodrugs.^16^

## Radiosynthesis and Characterization of [^195m^Pt]­azoPt

The synthesis of [^195m^Pt]­azoPt required modification of the cumbersome, multistep route to azoPt^13^ to develop a rapid, streamlined method compatible with radiochemical workflows and half-life of ^195m^Pt (see the Supporting Information (SI) for experimental details). Starting from [^195m^Pt]­Na_2_PtCl_6_ (13.1 mg, 426 MBq), reduction to Pt­(II) enabled sequential substitution with pyridine and azide to afford the *trans*-diazido Pt­(II) intermediate, followed by oxidation to yield [^195m^Pt]­azoPt (Figure 1A). On the scale used, precipitation of the Pt­(II) intermediate allowed in-vessel washing by resuspension/centrifugation, enabling the whole reaction to be performed within a single reaction vessel within 9 h. Dim-light conditions were employed to avoid photoactivation. The final product (50.6 MBq) was purified through solid-phase extraction which enabled direct formulation in ethanol, suitable for biological evaluation, with a (radio)­chemical yield of 12% based on [^195m^Pt]­Na_2_PtCl_6_, comparable to that of the nonradioactive route and sufficient for preclinical evaluation.

RP-HPLC analysis showed a UV trace of [^195m^Pt]­azoPt identical to that of the nonradioactive reference compound, with a single radioactive species coeluting with the UV peak (Figure 1B), confirming both chemical identity and high radiochemical purity. ESI–MS showed the same molecular ion and dimer peaks as nonradioactive azoPt (Figure S4), while the UV–vis spectrum retained the characteristic Pt←N_3_ charge-transfer band at 295 nm, which decreased upon blue-light irradiation, consistent with loss of Pt­(IV)-azide coordination on photoactivation (Figure S5).^13^ Together, these data demonstrate that metal-centered radiolabeling preserves both the chemical identity and functional photoreactivity of this Pt­(IV) prodrug, enabling direct interrogation of the intact platinum center without structural modification.

The modest radiochemical yield obtained arises largely from losses during the final purification steps required to remove primarily nonradioactive impurities. HPLC analysis of the crude reaction mixture (Figure S6) shows that [^195m^Pt]­azoPt is the major radioactive species, with only minor additional peaks observed.

## Stability and Behavior in Biological Media

Despite its susceptibility to photoreduction upon visible-light irradiation, [^195m^Pt]­azoPt remained chemically stable under the γ-emissions associated with ^195m^Pt decay with no radiolysis observed over 24 h in ethanol or PBS as confirmed by radio-HPLC analysis (Figures S7 and S8), demonstrating that incorporation of ^195m^Pt does not compromise the dark stability of azoPt. Size-exclusion chromatography after incubation in human serum showed retention of small-molecule behavior by [^195m^Pt]­azoPt, indicating minimal (1.5%) association with serum proteins (Figure 2A). Reverse-phase radio-HPLC analysis of low-molecular-weight platinum species after 2 h incubation of [^195m^Pt]­azoPt in serum and urine revealed a single peak with an elution time matching that of the intact compound under dark conditions, whereas light exposure produced multiple radioactive species (Figure 2B). Attempts to identify these species by mass spectrometry were unsuccessful, likely reflecting the complexity of photoactivation in biological media.^17^

**Figure 2:**
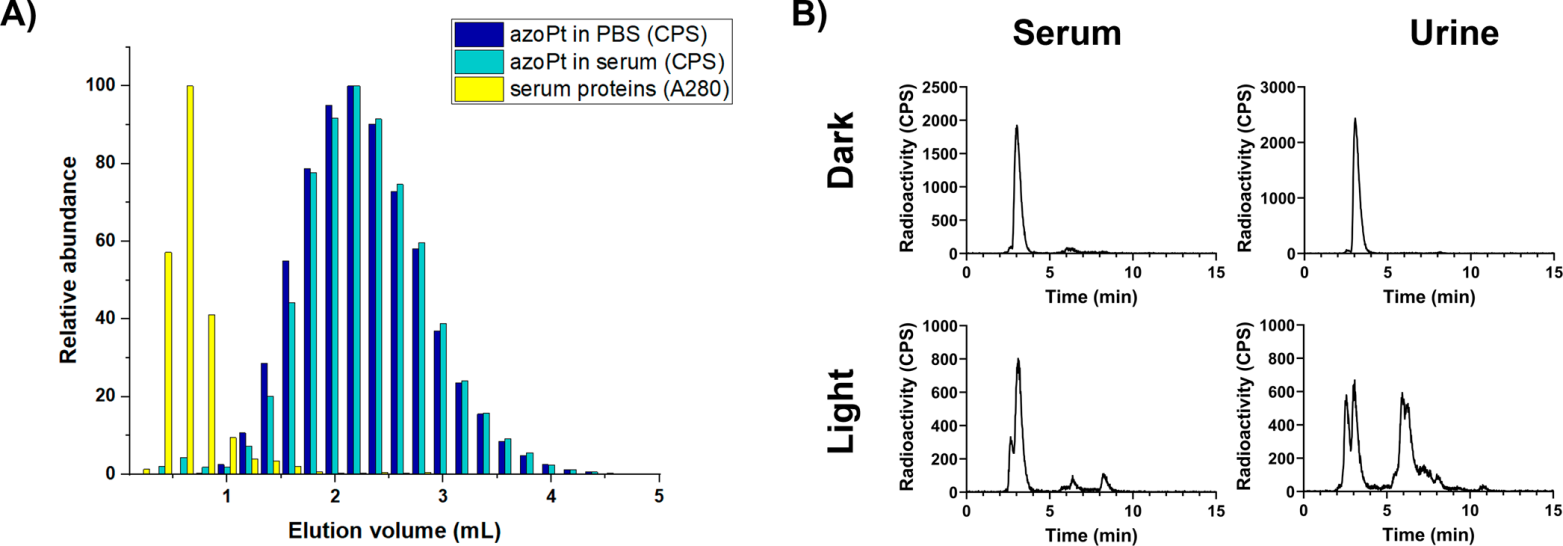
Stability of [^195m^Pt]­azoPt in biological media. (A) Size-exclusion chromatograms following incubation of [^195m^Pt]­azoPt in human serum (light blue) or PBS (dark blue), showing minimal (1.5%) association with serum proteins, which elute in less than 1 mL (yellow). (B) Reverse-phase radio-HPLC analysis of human serum (left) and urine (right) after incubation with [^195m^Pt]­azoPt in the dark or following blue-light irradiation, demonstrating preservation of the intact complex under dark conditions and formation of multiple photoproducts upon photoactivation.

## In Vivo Imaging and Biodistribution

Whole-body SPECT/CT imaging was used to visualize [^195m^Pt]­azoPt distribution in vivo compared to [^195m^Pt]­cisplatin,^18^ a clinically established benchmark whose pharmacokinetic behavior is dominated by renal elimination. Following intravenous administration in healthy mice (1.9–3.4 MBq, 13–24 mg kg^–1^), [^195m^Pt]­azoPt showed rapid clearance from the bloodstream with predominantly renal excretion, typical of small, hydrophilic molecules, and a minor hepatobiliary contribution. At 24 h postinjection, however, residual activity was unexpectedly localized in the bladder wall (Figure 3 and Figure S11). Mice administered [^195m^Pt]­cisplatin (2.5–2.8 MBq, 7–8 mg kg^–1^) exhibited similar early clearance, but diffuse residual uptake in liver and kidneys at 24 h postinjection, without bladder-specific retention, indicating that the bladder localization observed for [^195m^Pt]­azoPt reflects compound-specific in vivo behavior rather than generic platinum handling.

**Figure 3:**
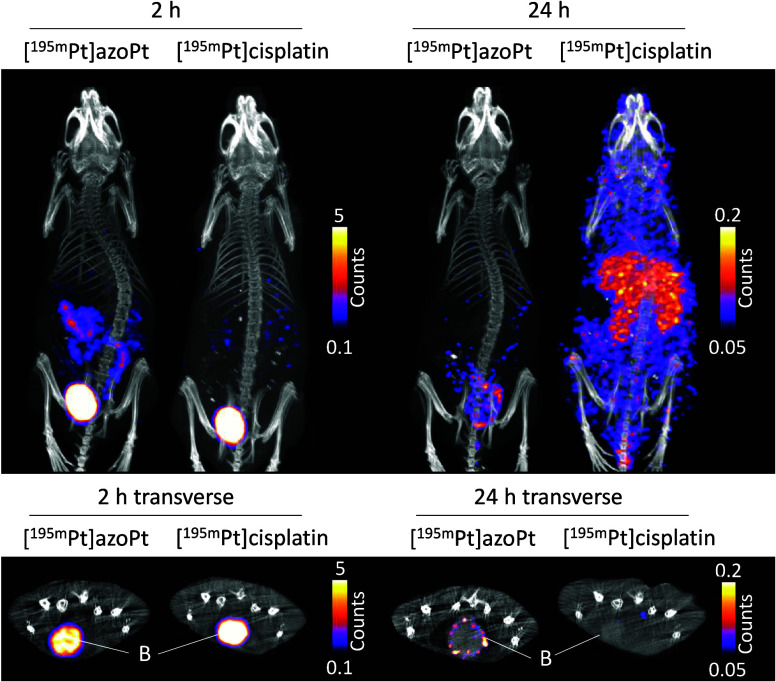
In vivo imaging in healthy mice. Longitudinal ^195m^Pt images of healthy mice following intravenous administration of [^195m^Pt]­azoPt or [^195m^Pt]­cisplatin at 2 and 24 h postinjection. Both compounds exhibit rapid renal clearance, but only [^195m^Pt]­azoPt displays persistent localization in the bladder wall at 24 h (visible in the transverse view) indicating compound-specific differences in biological behavior.

In mice bearing A2780 ovarian cancer xenografts, a clinically relevant model for platinum-based therapy, [^195m^Pt]­azoPt displayed low tumor uptake consistent with rapid renal clearance (Figure 4A), in line with reports of low tumor accumulation for cisplatin in similar models.^9^ Pronounced accumulation in the bladder wall was again observed and confirmed quantitatively by the ex vivo biodistribution (Figure 4B). Uptake values of 71 ± 23%ID g^–1^ at 2 h and 36 ± 10%ID g^–1^ at 24 h revealed persistent retention in bladder tissue. This suggests that bladder wall-associated activity was already present 2 h after injection, although it could not be distinguished from the intense signal of radioactive urine in the in vivo images.

**Figure 4:**
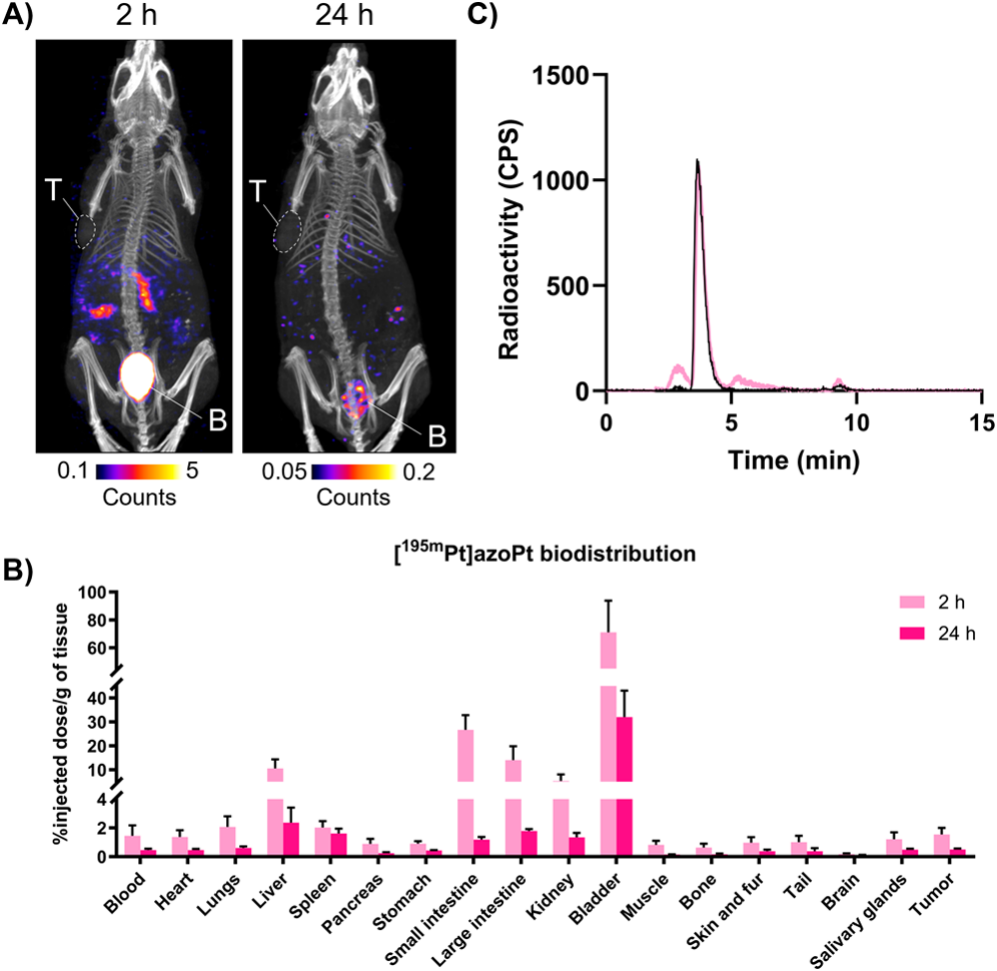
Bladder accumulation and in vivo stability of [^195m^Pt]­azoPt in tumor-bearing mice. (A) SPECT images of mice bearing A2780 ovarian cancer xenografts at 2 and 24 h following intravenous administration of [^195m^Pt]­azoPt, showing rapid clearance and pronounced localization in the bladder region. (B) Ex vivo biodistribution of [^195m^Pt]­azoPt at 2 and 24 h postinjection confirming high accumulation in bladder tissue. (C) Representative radio-HPLC chromatogram of urine collected 2 h postinjection (pink trace), compared with [^195m^Pt]­azoPt (black trace), identifying the intact agent as the predominant radioactive species.

Radio-HPLC analysis of urine collected 2 h postinjection (Figure 4C and Figure S13) showed intact [^195m^Pt]­azoPt as the predominant species (87 ± 3%), with only minor additional peaks detected, confirming high in vivo stability of the prodrug. While the predominance of [^195m^Pt]­azoPt in urine suggests that the intact compound may contribute to accumulation in the bladder wall, retained species may differ in composition, with partial transformation in the urinary or tissue environment potentially contributing to retention. Definitive speciation in tissues might benefit in future work from application of e.g. advanced mass spectrometry imaging techniques.

AzoPt and derivatives have previously shown high photocytotoxicity in bladder cancer cell lines, supporting the therapeutic relevance of this organ-specific accumulation.^19^

Notably, this distinctive bladder-specific distribution was not observed for a structurally modified Pt-succ-DFO–^68^Ga analogue,^20^ underscoring how derivatization required for ^68^Ga radiolabeling can mask intrinsic pharmacokinetic behavior, and illustrating the unique ability of metal-centered radiolabeling to reveal the authentic in vivo fate of platinum drugs, and specifically that of the platinum center itself.

The in vivo imaging data presented here reveal biological behavior of a photoactivatable platinum prodrug that cannot be captured by surrogate labeling strategies and would be difficult to uncover using bulk elemental analysis alone, which relies on prior tissue selection. The marked and persistent accumulation of [^195m^Pt]­azoPt in bladder tissue identifies this photoactivatable Pt­(IV) prodrug as a promising candidate for further investigation in the context of bladder photochemotherapy, a modality that is already undergoing clinical evaluation for nonmuscle-invasive bladder cancer with the ruthenium-based photodynamic agent TLD1433.^1^

More broadly, the successful ^195m^Pt radiolabeling of azoPt underscores the wider potential of platinum-centered radiolabeling for interrogating the pharmacokinetics and biodistribution of novel platinum drug candidates.

Recent advances in preclinical imaging instrumentation and radiometal availability—including radioisotopes of Ru, Re and Lu, commonly used in metal-based photoactive agents—now make metal-centered radiolabeling a practical approach that removes the need for structural modification and provides a powerful tool to accelerate metallodrug discovery and translation.^21,22^ Half a century after the first studies with [^195m^Pt]­cisplatin, radionuclide imaging is now ready to stimulate a new era in medicinal inorganic chemistry.

## Supplementary Material



## Notes

Material and methods, experimental procedures, radionuclide purity table, and supplementary figures (PDF)

## References

[1] Monro S., Colón K. L., Yin H. M., Roque J., Konda P., Gujar S., Thummel R. P., Lilge L., Cameron C. G., McFarland S. A. (2019). Transition Metal Complexes and Photodynamic Therapy
from a Tumor-Centered Approach: Challenges, Opportunities, and Highlights
from the Development of TLD1433. Chem. Rev..

[2] Wang Z., Wang N., Cheng S.-C., Xu K., Deng Z., Chen S., Xu Z., Xie K., Tse M.-K., Shi P., Hirao H., Ko C.-C., Zhu G. (2019). Phorbiplatin, a Highly
Potent Pt­(IV) Antitumor Prodrug That Can Be Controllably Activated
by Red Light. Chem..

[3] Arora K., Herroon M., Al-Afyouni M. H., Toupin N. P., Rohrabaugh T. N., Loftus L. M., Podgorski I., Turro C., Kodanko J. J. (2018). Catch and
Release Photosensitizers: Combining Dual-Action Ruthenium Complexes
with Protease Inactivation for Targeting Invasive Cancers. J. Am. Chem. Soc..

[4] Zhang L., Wang P., Zhou X.-Q., Bretin L., Zeng X., Husiev Y., Polanco E. A., Zhao G., Wijaya L. S., Biver T., Le Devedec S. E., Sun W., Bonnet S. (2023). Cyclic Ruthenium–Peptide
Conjugates as Integrin-Targeting Phototherapeutic Prodrugs for the
Treatment of Brain Tumors. J. Am. Chem. Soc..

[5] An J., Lv K.-P., Chau C. V., Lim J. H., Parida R., Huang X., Debnath S., Xu Y., Zheng S., Sedgwick A. C., Lee J. Y., Luo D., Liu Q., Sessler J. L., Kim J. S. (2024). Lutetium Texaphyrin–Celecoxib
Conjugate as a Potential Immuno-Photodynamic Therapy Agent. J. Am. Chem. Soc..

[6] Redrado M., Acharya S., Mesdom P., Babu T., Southwell J. W., Oliveira L. S., Hidalgo S., Arnoux P., Frochot C., Gibson D., Gasser G. (2025). A Triple Threat Against Ovarian Cancer:
Os­(II)–Pt­(IV)–Ceritinib Conjugates for Photodynamic
Therapy, Chemotherapy, and Immunogenic Cell Death Induction. Angew. Chem., Int. Ed..

[7] Aalbersberg E. A., de Wit-van der Veen B. J., Zwaagstra O., Codée-van der Schilden K., Vegt E., Vogel W. V. (2017). Preclinical
Imaging Characteristics and Quantification of Platinum-195m SPECT. Eur. J. Nucl. Med. Mol. Imaging.

[8] Smith P. H. S., Taylor D. M. (1974). Distribution and
Retention of Antitumor Agent Pt-195m-Cis-Dichlorodiammineplatinum­(II)
in Man. J. Nucl. Med..

[9] de
Roest R. H., van Walsum M. S., van der Schilden K., Brakenhoff R. H. (2024). Pharmacodynamics and Biodistribution of ^195m^Pt-Cisplatin (CISSPECT®) in Head and Neck Squamous Cell Carcinoma. EJNMMI Res..

[10] Hoogenkamp D. S., de Wit-van der Veen B.
J., Hendriksen J., van der Schilden K., Nanne J. A. M., Belderbos J. S. A., Rossi M. M., Mooijer M. P. J., Funke U., Hendrikse N. H., Vogel W. V., Aalbersberg E. A. (2025). [^195m^Pt]­Cisplatin for
Lung Cancer Imaging: A Pilot Study. EJNMMI Res..

[11] Nadar R. A., Farbod K., Codée-van
der Schilden K., Schlatt L., Crone B., Asokan N., Curci A., Brand M., Bornhäuser M., Iafisco M., Margiotta N., Karst U., Heskamp S., Boerman O. C., van den
Beucken J., Leeuwenburgh S. C. G. (2020). Targeting of Radioactive Platinum–Bisphosphonate
Anticancer Drugs to Bone of High Metabolic Activity. Sci. Rep..

[12] Shi H. Y., Imberti C., Sadler P. J. (2019). Diazido Platinum­(IV)
Complexes for
Photoactivated Anticancer Chemotherapy. Inorg.
Chem. Front..

[13] Farrer N.
J., Woods J. A., Salassa L., Zhao Y., Robinson K. S., Clarkson G., Mackay F. S., Sadler P. J. (2010). A Potent Trans-Diimine
Platinum Anticancer Complex Photoactivated by Visible Light. Angew. Chem., Int. Ed..

[14] Shi H. Y., Carter O. W. L., Ponte F., Imberti C., Gomez-Gonzalez M. A., Cacho-Nerin F., Quinn P. D., Parker J. E., Sicilia E., Huang H. Y. (2024). A Photodynamic and Photochemotherapeutic Platinum-Iridium
Charge-Transfer Conjugate for Anticancer Therapy. Angew. Chem., Int. Ed..

[15] Shi H. Y., Ponte F., Grewal J. S., Clarkson G. J., Imberti C., Hands-Portman I., Dallmann R., Sicilia E., Sadler P. J. (2024). Tuning
the photoactivated anticancer activity of Pt­(IV) compounds via distant
ferrocene conjugation. Chem. Sci..

[16] Johnstone T. C., Suntharalingam K., Lippard S. J. (2016). The Next Generation of Platinum Drugs:
Targeted Pt­(II) Agents, Nanoparticle Delivery, and Pt­(IV) Prodrugs. Chem. Rev..

[17] Shi H. Y., Ward-Deitrich C., Ponte F., Sicilia E., Goenaga-Infante H., Sadler P. J. (2024). Photosubstitution and photoreduction
of a diazido platinum­(IV)
anticancer complex. Dalton Trans..

[18] Hoeschele J. D., Butler T. A., Roberts J. A., Guyer C. E. (1982). Analysis and Refinement
of the Microscale Synthesis of the ^195m^Pt-Labeled Antitumor
Drug, cis-Dichlorodiammineplatinum­(II). Radiochim.
Acta.

[19] Shi H., Clarkson G. J., Sadler P. J. (2024). Tuning the Phototherapeutic Activity
of Pt­(IV) Complexes for Bladder Cancer via Modification of trans N-Heterocyclic
Ligands. Inorg. Chem. Front..

[20] Imberti C., Lok J., Coverdale J. P. C., Carter O. W. L., Fry M. E., Postings M. L., Kim J., Firth G., Blower P. J., Sadler P. J. (2023). Radiometal-Labeled
Photoactivatable Pt­(IV) Anticancer
Complex for Theranostic Phototherapy. Inorg.
Chem..

[21] Imberti C., Blower P. J. (2025). Roadmap of Opportunities and Challenges for Metal Chemistry
in Molecular Imaging: From Gamma Camera Imaging to PET and Multimodality
Imaging. Adv. Inorg. Chem..

[22] Holland, J. P. The Radiopharmaceutical Chemistry of Seldom-Used Radionuclides in Nuclear Medicine. In Radiopharmaceutical Chemistry; Lewis, J. S. , Windhorst, A. D. , Zeglis, B. M. , Eds.; Springer: 2019; pp 425–446.

