# Supplementary material for: In Vivo Imaging of a Photoactivatable Platinum Prodrug by Metal-Centered Radiolabeling
Source: J Am Chem Soc. 2026 May 30;148(22):22413–7. doi: 10.1021/jacs.6c05292 (PMC13266692; doi:10.1021/jacs.6c05292)
Supplement: Supplementary file 1 [file ja6c05292_si_001.pdf]

Supporting Information

In Vivo Imaging of a Photoactivatable Platinum Prodrug by Metal-Centered Radiolabeling

George Firth[1]†, Jana Kim[1]†, Huayun Shi[2], Kavitha Sunassee[1], Oene Zwaagstra[3], Karlijn Van der Schilden[3], Philip J. Blower[1], Peter J. Sadler[2]* and Cinzia Imberti[1,2]*.

1 School of Biomedical Engineering and Imaging Sciences, King's College London, London, UK
2 Department of Chemistry, University of Warwick, Coventry, UK
3 NRG PALLAS, Petten, Netherlands

† These authors equally contributed to the manuscript.

*** Emails**: p.j.sadler@warwick.ac.uk, cinzia.imberti@kcl.ac.uk

Table of Contents

Abbreviations

Material and Methods

Experimental Procedures

Traditional synthetic route for azoPt (*trans,trans,trans*-[$Pt(OH)_2(N_3)_2(pyridine)_2$])

Optimized procedure toward ^{195m}Pt radiolabeling

Radiosynthesis and purification of [^{195m}Pt]azoPt

Stability to X-ray irradiation

Interaction with human serum proteins

Stability in human serum and urine

In vivo studies in healthy mice

In vivo studies in A2780 ovarian cancer xenografts

Table S1. Exemplar radionuclide purity of [^{195m}Pt]Na_2PtCl_6 batch

Figure S1. Dose calibrator measurement of a known quantity of ^{195m}Pt

Figure S2. HPLC analysis (Method 1) of azoPt before and after purification

Figure S3. HPLC analysis (Method 2) of [^{195m}Pt]azoPt

Figure S4. ESI-MS spectra of [^{195m}Pt]azoPt and azoPt

Figure S5. UV-vis spectra of [^{195m}Pt]azoPt

Figure S6. HPLC analysis of crude [^{195m}Pt]azoPt

Figure S7. HPLC analysis of [^{195m}Pt]azoPt after 24 h in ethanol

Figure S8. HPLC analysis of [^{195m}Pt]azoPt after 24 h in PBS

Figure S9. HPLC analysis of [^{195m}Pt]azoPt after an X-ray CT scan

Figure S10. HPLC analysis of [^{195m}Pt]cisplatin

Figure S11. [^{195m}Pt]azoPt *ex vivo* biodistribution in healthy mice

Figure S12. A2780 tumor growth curve

Figure S13. HPLC analysis of the urine from mice injected with [^{195m}Pt]azoPt

References

Abbreviations

%ID	percent injected dose
azoPt	*trans,trans,trans*-[Pt(OH)$_2$(N$_3$)$_2$(pyridine)$_2$]
CPS	counts per second
CT	computed tomography
ESI	electrospray ionization
FA	formic acid
LMCT	ligand-to-metal charge transfer
PBS	phosphate buffered saline
RCF	relative centrifugal force
RP-HPLC	reverse-phase high-performance liquid chromatography
RT	room temperature
SD	standard deviation
SPE	solid phase extraction
SPECT	single-photon emission computed tomography
TIC	total ion current
TLC	thin layer chromatography
$t_{1/2}$	half-life

Materials and Methods

K_2PtCl_4 and Na_2PtCl_6 were obtained from Acros Organics. Pyridine was purchased from Fisher Scientific. All other chemicals were obtained from Sigma Aldrich, unless otherwise specified, and used without further purification. [^{195m}Pt]Na_2PtCl_6 was produced in the High Flux Reactor of NRG PALLAS (Petten, Netherland) as previously described[1-3] with specific activity of ≈ 180 MBq ^{195m}Pt/mg Pt at end of irradiation (Table S1, Figure S1). [^{195m}Pt]cisplatin was prepared by NRG PALLAS and Amsterdam University Medical Center according to published procedures[1-3]. Its identity and purity were confirmed by HPLC (Figure S10) in accordance with the European Pharmacopoeia, Cisplatin monograph (Ph. Eur. monograph 0599).

Human serum was harvested from blood obtained from healthy donors using gold top tubes for serum separation (with clotting activator and separation gel, Medisave). Tubes were incubated at RT for 30 min and centrifuged at 22 °C, 1500 RCF for 10 min before removing the serum (supernatant) from the pelleted cells, followed by filtration through a 0.45 µm filter. Human urine was obtained from healthy donors and passed through a 0.45 µm filter.

Initial analytical RP-HPLC studies at the University of Warwick were carried out on an Agilent 1100 HPLC equipped with an Agilent ZORBAX Eclipse XDB-C18 column (250×4.6 mm, 5 µm, flow rate: 1 mL min^{-1}), using water (solvent A) and ethanol (solvent B) as mobile phases and gradient: 0-2 min 2% B, 2-35 min ramp to 50% B, 36-40 min 2% B (Method 1).

HPLC and radio-HPLC investigation at KCL were performed on an Agilent 1200 LC system with UV detection at 214 or 254 nm coupled to a LabLogic Flow-Count radioactivity detector with a sodium iodide probe (B-FC-3200), using an Agilent Eclipse XDB-C18 column (150x4.6 mm, 5 µm) with a 1 mL min^{-1} flow rate. Two methods were used on this system:

- Method 2**:** Mobile phase: water (solvent A) and ethanol (solvent B), gradient: 0-2 min 2% B, 2-25 min ramp to 50% B, 26-30 min 2% B.
- Method 3: Mobile phase: water with 0.1% FA (solvent A), acetonitrile with 0.1% FA (solvent B). Gradient:0-2 min 5% B, 2-11 min ramp to 95% B, 12-15 min 5% B.

Mass spectrometry was carried out on an Advion expression compact ESI-MS.

UV-vis spectroscopy was performed using a Perkin-Elmer Lambda 365 UV/vis spectrophotometer and 1-cm quartz cuvettes. An in-house developed LED-light source (4.8 mW cm^{-2}, 465 nm) was utilized for photoactivation studies with blue light.

Radioactivity was measured on a Capintec CRC-25R (for activities greater than 0.5 MBq, Figure S1) or on a LKB-Wallac CompuGamma 1282 Gamma Counter (for activities below 0.5 MBq), after calibrating both instruments with known quantities of ^{195m}Pt.

SPECT/CT scans were acquired using a NanoSPECT/CT Silver Upgrade 4-head scanner with 4 × 9 (1.4 mm) pinhole collimators (Mediso Ltd., Budapest, Hungary). A double energy window of 66.8 keV ± 20% and 98.9 keV ± 20% and helical scanning mode were used. CT images were acquired with a 55 kVp X-ray source, 1000 ms exposure time in 180 projections over approximately 9 min. CT images were reconstructed in Nucline (v2.00) at 0.16 × 0.16 × 0.16 mm voxel size. SPECT images were reconstructed at 0.3 mm isotropic voxel size using HiSPECT (ScivisGmbH), a reconstruction software package.

Experimental Procedures

Synthetic route for azoPt (*trans,trans,trans*-[$Pt(OH)_2(N_3)_2(pyridine)_2$])

Caution! *Heavy-metal azides can be shock-sensitive detonators. We did not encounter any problems during the work reported here, but due care and attention with appropriate precautions should be taken in their synthesis and handling.*

Synthesis was performed according to modified published procedures[4] and carried out in the dark to minimize light activation. K_2PtCl_4 (200 mg) was dissolved in the minimum volume of water, stirred, and heated to reflux for 1 h following addition of pyridine (20 eq). An aqueous solution of sodium azide (20 eq) was then added dropwise under reflux, and the mixture was stirred for a further 2 h. The resulting yellow precipitate was collected by filtration and washed with cold water (3×) and ethanol (3×), then air-dried.

The solid was transferred to a round-bottom flask and treated with H_2O_2 (30% v/v, 10 mL). The suspension was stirred overnight at 60 °C, filtered, and the filtrate freeze-dried to yield a crude yellow product. Purification by silica column chromatography (CH_2Cl_2/MeOH gradient), monitored by TLC, afforded the pure complex (55% yield).

Optimization of procedures for ^{195m}Pt radiolabeling

The procedure was redesigned to be compatible with radiolabeling — minimizing handling, reducing reaction time and using single-use vessels. Since ^{195m}Pt is provided as [^{195m}Pt]Na_2PtCl_6, synthesis was started from 20 mg Na_2PtCl_6 (scale consistent with the Pt mass expected from reported specific activities). Reagent molar equivalents were reduced to 10 eq pyridine and 12 eq sodium azide to simplify purification without increasing byproduct formation. All steps were conducted in a microcentrifuge tube.

Na_2PtCl_6 was dissolved in 200 µL of water followed by addition of aqueous hydrazine (0.25 M, 85 µL, 0.5 eq). The solution turned from yellow to dark orange and was heated for 10 min at 45 °C and then 15 min at 100 °C. The vial was opened and evaporated to dryness under nitrogen. The residue was dissolved in water (125 µL) and evaporated twice more before being redissolved in water (100 µL, pH ≈ 1). Pyridine was then added until the solution reached pH 6 (35 µL, 10 eq), and the sealed tube heated at 95 °C for 1 h. After cooling, sodium azide was added, and the reaction heated at 95 °C for a further 2 h. The resulting yellow Pt(II) precipitate was isolated by centrifugation (3 min at 100 RCF) and washed with cold water (3×) and ethanol (3×). The solid was dried under a nitrogen stream and 600–1000 µL of H_2O_2 (30% v/v) added. A vented cap (lid fitted with a needle) was used to allow gas exchange while minimizing contamination. The mixture was heated at 65 °C for 2 h; if solids remained after 1 h, an additional 500 µL H_2O_2 was added.

Reaction completion was monitored by HPLC (Method 1), which showed a major peak at 16 min corresponding to the desired Pt(IV) product, as well as byproducts peaks (Figure S2 top panel). The crude mixture was passed through a Sep-Pak Light C18 cartridge, and fractions (10 × 0.5 mL) analyzed by ESI-MS. Fractions containing product were combined, loaded onto a Sep-Pak Short C18 cartridge, washed with water (5 × 0.5 mL), and eluted with ethanol. Evaporation under nitrogen afforded the purified complex (15% yield), which was confirmed by HPLC to be free from the byproducts observed in the crude mixture (Figure S2 bottom panel).

Radiosynthesis and purification of [^{195m}Pt]azoPt

[^{195m}Pt]Na_2PtCl_6 (13.1 mg, 28.8 µmol, 426 MBq at the start of synthesis) was dissolved in water (400 µL) to give a yellow solution and transferred to a microcentrifuge vial. The volume was reduced to 200 µL under a gentle nitrogen stream at 90 °C, followed by addition of hydrazine (0.25 M, 55 µL, 0.5 eq). After the solution turned dark orange, it was heated as above and then evaporated to dryness under nitrogen. The residue was dissolved in water (125 µL) and evaporated twice more before final redissolution.

Pyridine (22 µL, 9.6 eq) was added, and the sealed vial was heated at 100 °C for 60 min. After cooling for 5 min, sodium azide (22.5 mg in 250 µL water, 12 eq) was added, and the mixture was heated for 2 h at 100 °C in the dark, forming a yellow precipitate. Water (0.8 mL) was added, and the mixture was centrifuged (3 min at 100 RCF). The solid was washed with cold water (3×) and ethanol (3×), centrifuging at 200 RCF each time. After drying under nitrogen, the measured activity was 329 MBq after 6 h (partial, non-decay-corrected yield = 81%).

Oxidation to the Pt(IV) product was carried out using the optimized protocol: H_2O_2 (30% v/v, 600 µL) was added, the vial sealed with a vented cap, and the mixture heated at 65 °C adding an extra 500 µL H_2O_2 if solids remained after 1 h. After 2 h, the crude mixture was analyzed by HPLC (Method 3), showing high radiochemical conversion in the radiochromatogram but presence of a prominent non-radioactive species in the UV trace (Figure S6). The crude mixture was passed through a Sep-Pak Light C18 cartridge (10 × 0.5 mL fractions), and those containing sufficient activity (118 MBq combined; partial, non-decay-corrected radiochemical yield at 8 h = 28%) were pooled. The combined fractions were loaded onto a Sep-Pak Short C18 cartridge, washed with water (5 × 0.5 mL) and eluted with ethanol (4 × 0.5 mL). The second ethanol fraction typically contained 80–85% of the eluted activity. A small aliquot was evaporated, redissolved in water and analyzed by two HPLC methods to confirm radiochemical purity (Method 3 in Figure 1 and Method 2 in Figure S3).

50.6 MBq of [^{195m}Pt]azoPt were obtained, corresponding to a 12.5% non-decay-corrected radiochemical yield (12.7% decay-corrected at 9 h). The remaining ethanol solution was stored at 4 °C, protected from light. For biological experiments, ethanol was evaporated at 70 °C under nitrogen and the residue was redissolved in PBS. Stability in ethanol and PBS was confirmed by HPLC up to 24 h (Figure S7 and Figure S8).

Stability to X-ray irradiation

Stability of [^{195m}Pt]azoPt to X-rays used for CT measurements was verified by performing a sham CT scan with the same parameters to be used for preclinical CT imaging. A PBS solution of [^{195m}Pt]azoPt was analyzed by RP-HPLC before and after the CT confirming stability to X-ray irradiation (Figure S9).

Interaction with human serum proteins

To quantify azoPt interaction with serum proteins in the absence of light irradiation, [^{195m}Pt]azoPt was dissolved in PBS (10 µL, 0.3 MBq) and incubated either with human serum from healthy volunteers (190 µL) or with PBS (190 µL) at 37 °C for 24 h. The mixture was then loaded onto a PD10 column, followed by elution with PBS, collection of 200 µL fractions and analysis using a γ-counter. A third vial, containing serum only (200 µL) was subjected to the same procedure (24 h at 37 °C, followed by PD10 elution) and the fraction analyzed on a nanodrop detector (ThermoFisher) measuring absorbance at 280 nm to determine protein concentration.

Stability in human serum and urine

The stability of [^{195m}Pt]azoPt in the presence of human serum and urine (from healthy volunteers), under dark conditions or light irradiation was also determined by RP-HPLC (Method 3).

For serum stability, [^{195m}Pt]azoPt in PBS (40 µL, ≈ 1.1 MBq) was added to 160 µL of serum and incubated for 2 h in the dark or for 1 h in the dark followed by 1 h of irradiation with blue light (465 nm, 4.8 mW cm^{-2}). Then 200 µL of acetonitrile was added to precipitate the serum proteins, the vial was centrifuged and the supernatant collected and evaporated. The residue was redissolved in 200 µL of water and injected into the HPLC (Method 3).

For urine stability, [^{195m}Pt]azoPt in PBS (30 µL, ≈ 0.8 MBq) was added to 170 µL of urine and incubated as previously described. After 2 h, the samples were injected into the HPLC (Method 3).

***In vivo* studies in healthy mice**

All *in vivo* experiments were carried out in accordance with British Home Office regulations governing animal experimentation and complied with guidelines on responsibility in the use of animals in bioscience research of the U.K. Research Councils and Medical Research Charities, under U.K. Home Office project and personal licenses.

For *ex vivo* biodistributions the whole organs (tail, heart, lungs, spleen, stomach, kidneys, bladder and, where present, tumor) were collected. The tibia was used as representative of bone. Skin and fur were collected from mouse ears and muscle was taken from the hindlimb. Only part of liver, and the small and large intestine (emptied of their contents, which were also measured) were collected and their radioactivity measured.

Six female BALB/c nude mice (7-9 weeks) were anesthetized with isoflurane, injected intravenously with [^{195m}Pt]azoPt (1.9-3.4 MBq in 100 µL PBS) and imaged for 2 hours either immediately after injection (4 mice, 0-2 h time point) or the day after (2 mice, 22-24 h time point). When imaging was performed immediately after injection, mice were kept under anesthesia for the whole procedure; when imaging was performed the following day, animals were allowed to recover after injection. At the end of the scan, mice were culled and the *ex vivo* biodistribution of [^{195m}Pt]azoPt was determined by organ gamma counting (Figure S11). An additional 2 mice were injected with [^{195m}Pt]azoPt and culled at 24 h to determine *ex vivo* biodistribution. Four mice were injected with [^{195m}Pt]cisplatin (2.5-2.8 MBq in 100 µL saline) and imaged at the same time points for comparison.

***In vivo* studies in A2780 ovarian cancer xenografts**

Eight female BALB/c nude mice (7-9 weeks) were subcutaneously injected with A2780 ovarian cancer cells (5 x 10^6 cells in 100 µL of PBS) on their right shoulder. Animals were monitored for weight and tumor growth (Figure S12). Imaging experiments were performed on mice with palpable tumors (> 150 mm^3 volume)

Three animals were injected intravenously with [^{195m}Pt]azoPt (3.3-3.5 MBq in 100 µL PBS) and imaged for 2 hours, then allowed to recover and imaged again at 22-24 h, followed by culling and *ex vivo* biodistribution by organ gamma counting. Four animals were injected intravenously with [^{195m}Pt]azoPt (2.1-4.1 MBq in 100 µL PBS) and sacrificed at 2 h for *ex vivo* biodistribution. Urine of these animals was analyzed by HPLC and relevant peaks collected and analyzed by ESI-MS (Figure 4 and Figure S13).

Table S1. Example of radionuclide purity for a batch of [^{195m}Pt]Na_2PtCl_6 at the end of irradiation.

Nuclide	Activity [MBq]	Percentage [%]	$t_{1/2}$
^{195m}Pt	1050	93.4	4.01 days
^{197}Pt	41	3.6	19.9 h
^{198}Au	0.9	0.08	2.69 days
^{199}Au	18	1.6	3.13 days
^{192}Ir	0.6	0.8	74 days
^{194}Ir	14	1.2	19 h

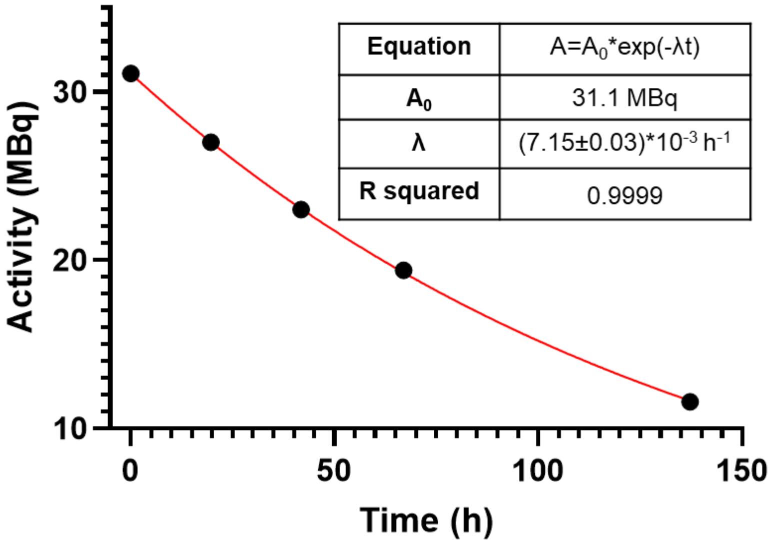

Figure S1 Decay curve of a known amount of ^{195m}Pt (31.1 MBq at time = 0 h) measured on a Capintec CRC-25R dose calibrator. The decay constant from radioactive decay fit: $\lambda = (7.15 \pm 0.03) \times 10^{-3}\ h^{-1}$, is consistent with reported half-life of ^{195m}Pt (4.01 days), confirming radionuclide purity at the start of the experiments.

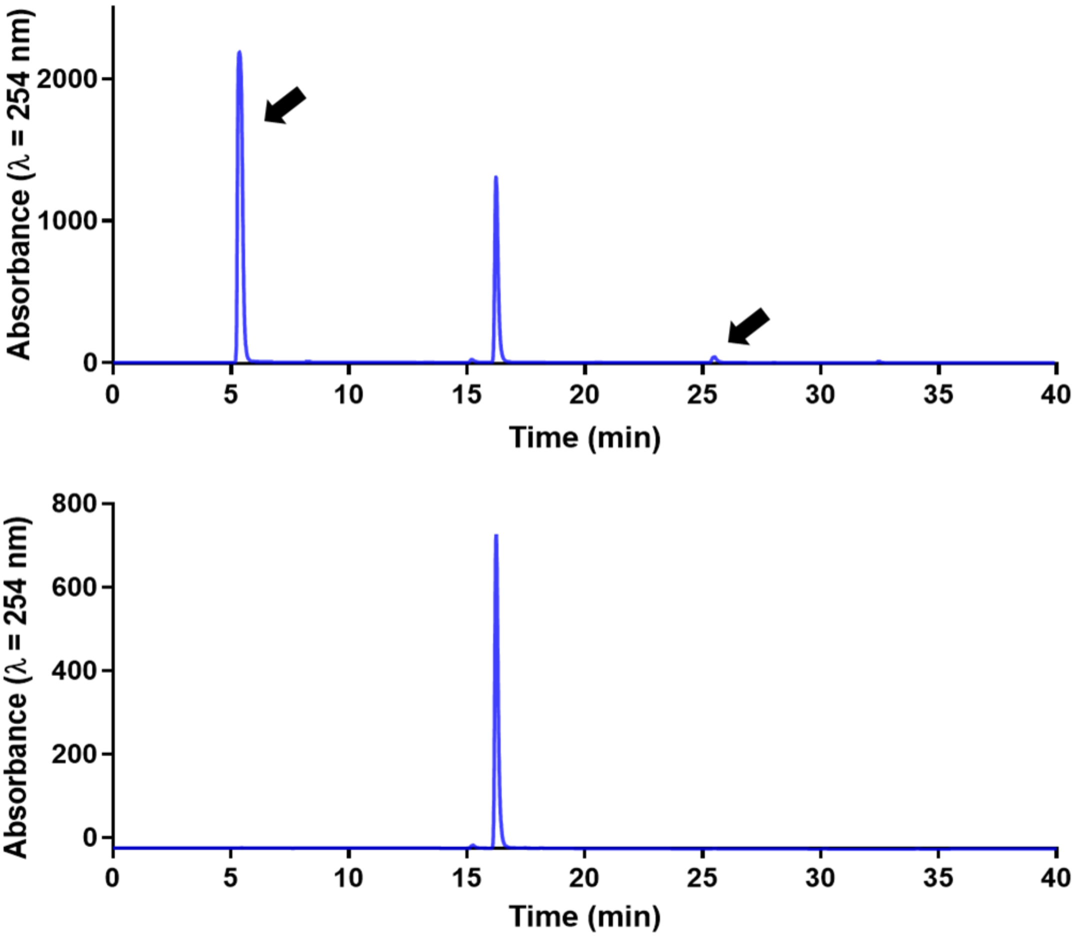

Figure S2. HPLC analysis of azoPt (Method 1) before and after SPE showing effective removal of impurities.

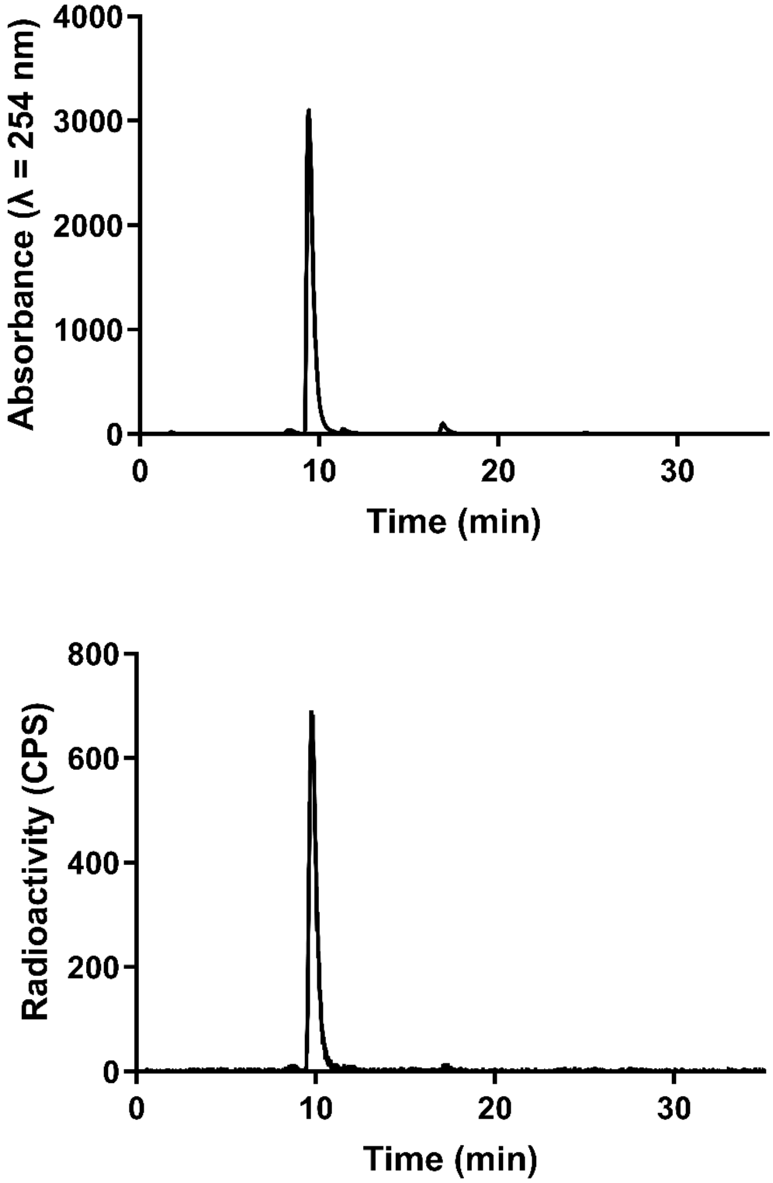

Figure S3. HPLC analysis of [^{195m}Pt]azoPt (Method 2) confirming purity of the radiolabeled complex.

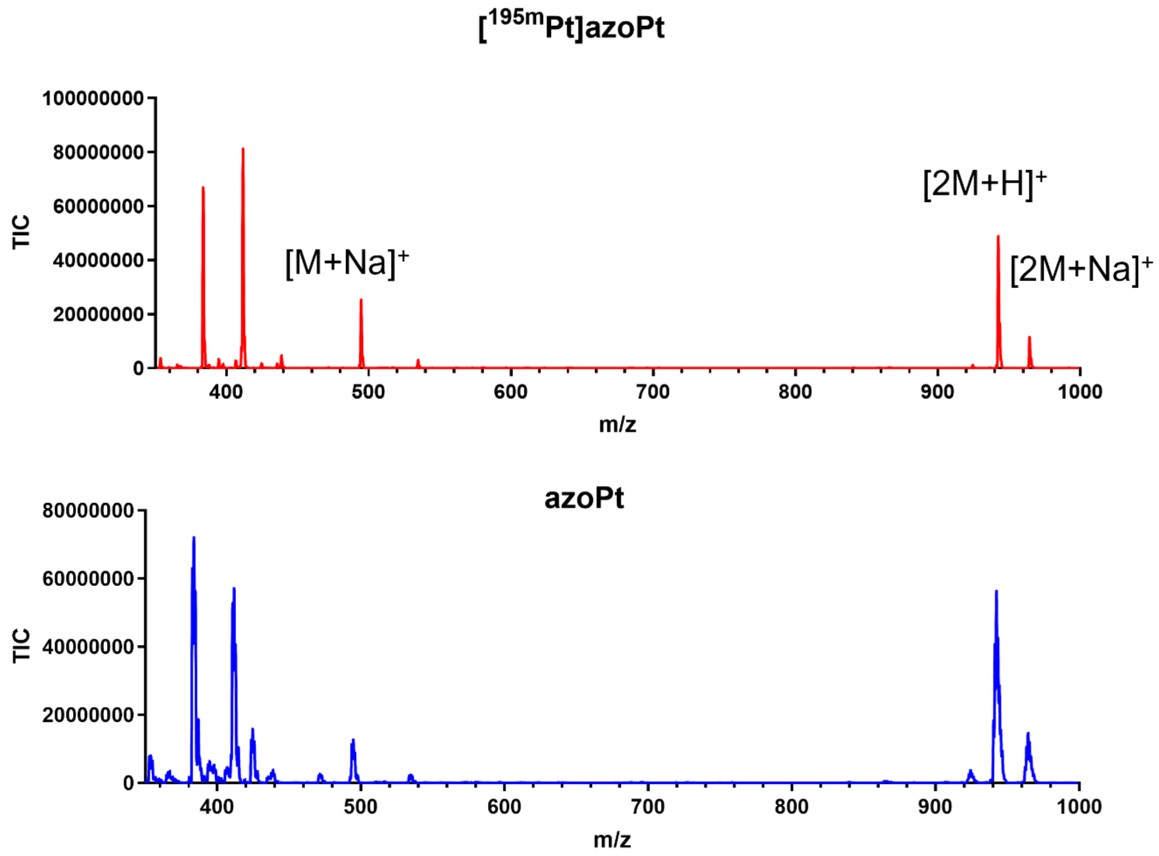

Figure S4. ESI-MS spectra of [^{195m}Pt]azoPt (red) and non-radioactive azoPt (blue) exhibiting the same fingerprint peaks for this complex including $[M+Na]^+$ = 494.8 m/z, $[2M+H]^+$ = 942.8 m/z and $[2M+Na]^+$ = 964.7 m/z).

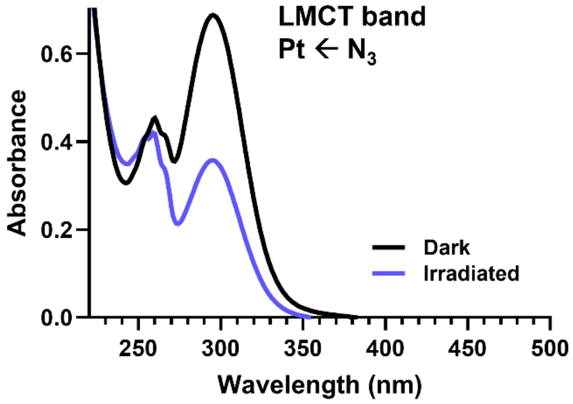

Figure S5. UV-vis spectra of [^{195m}Pt]azoPt showing the characteristic LMCT band at 295 nm, which decreases in intensity upon irradiation.

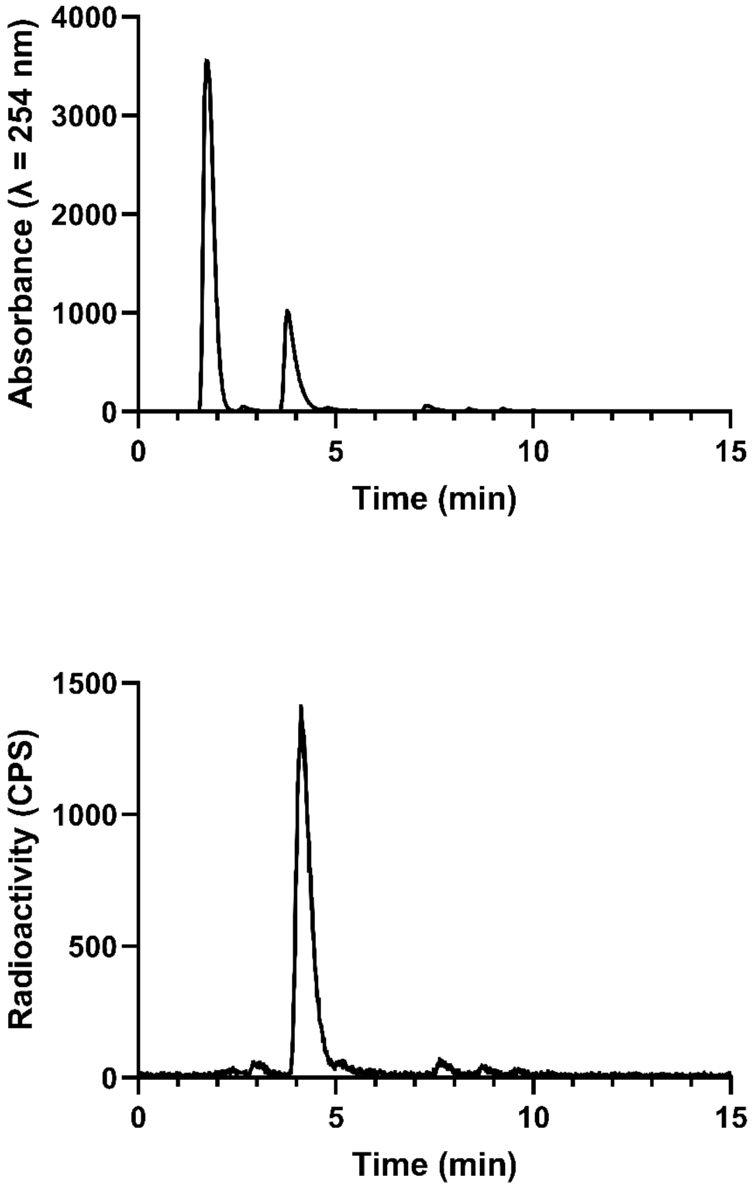

Figure S6. HPLC analysis of crude [^{195m}Pt]azoPt (Method 3). The UV chromatogram shows a major non-radioactive species, while the radiochromatogram indicates [^{195m}Pt]azoPt as the main radioactive peak, with minor additional radioactive components.

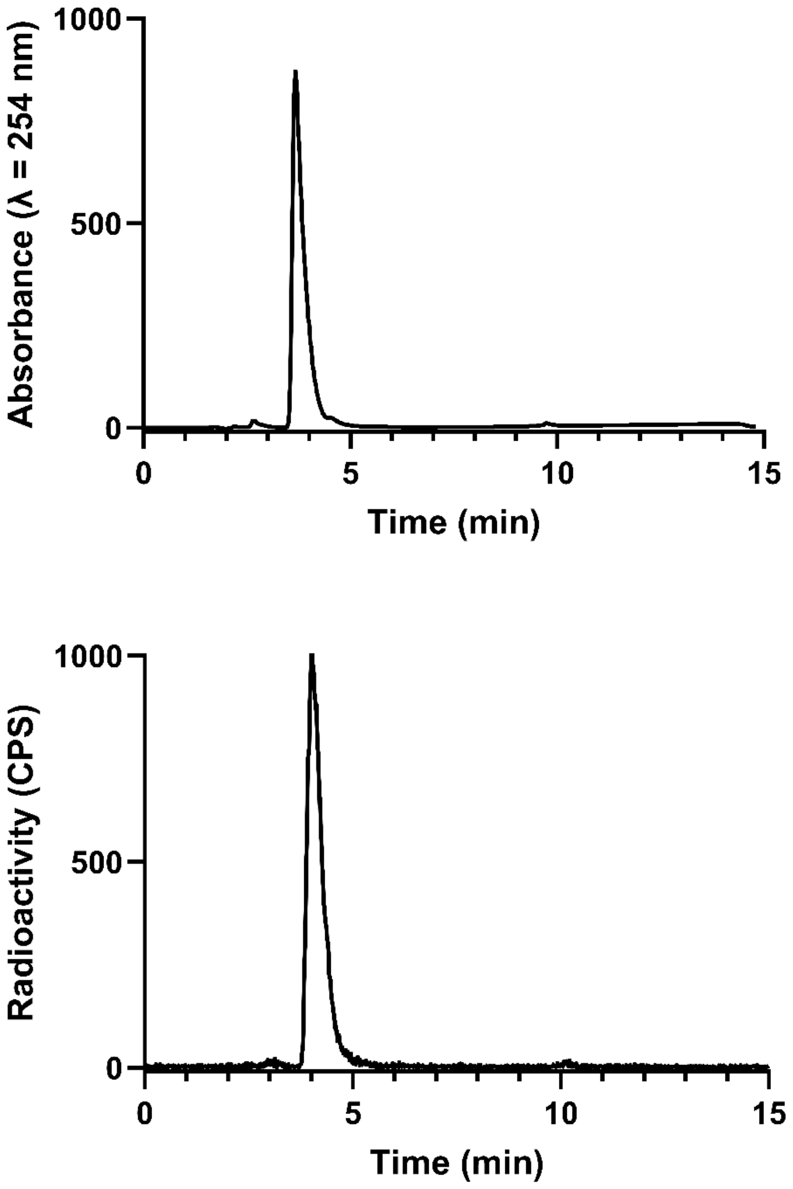

Figure S7. HPLC analysis of [^{195m}Pt]azoPt (Method 3) after 24 h in ethanol.

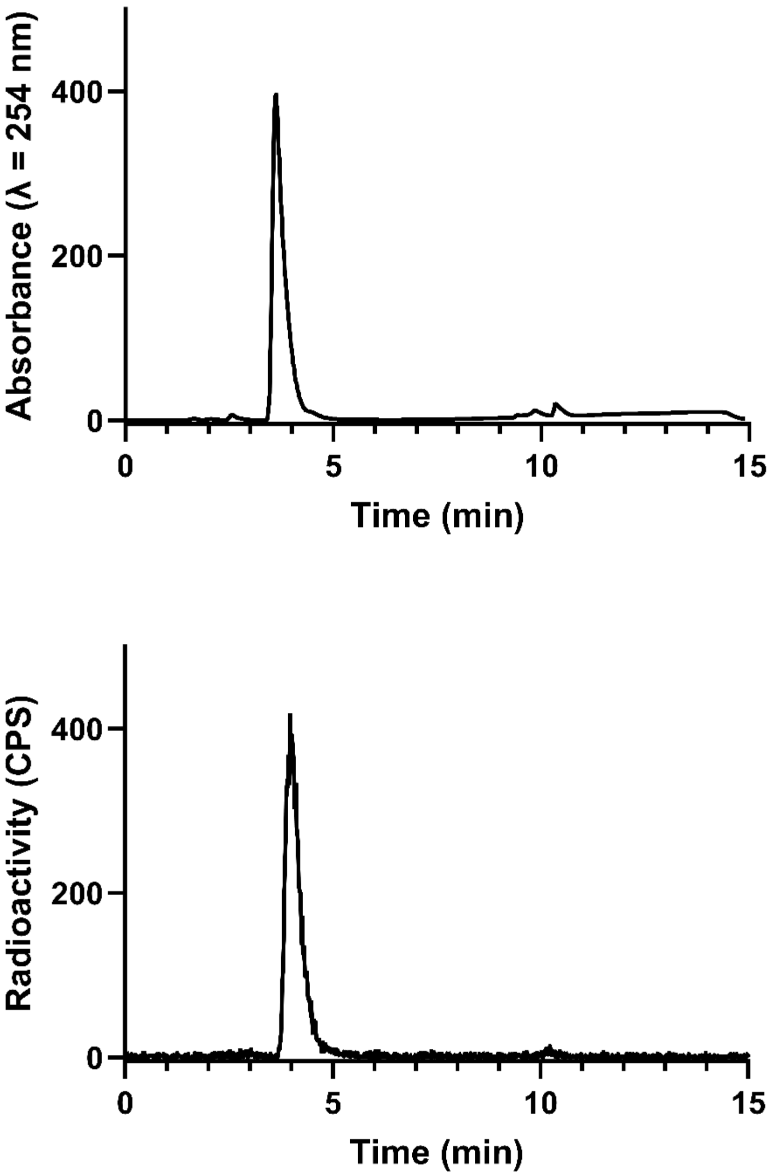

Figure S8. HPLC analysis of [^{195m}Pt]azoPt (Method 3) after 24 h in PBS.

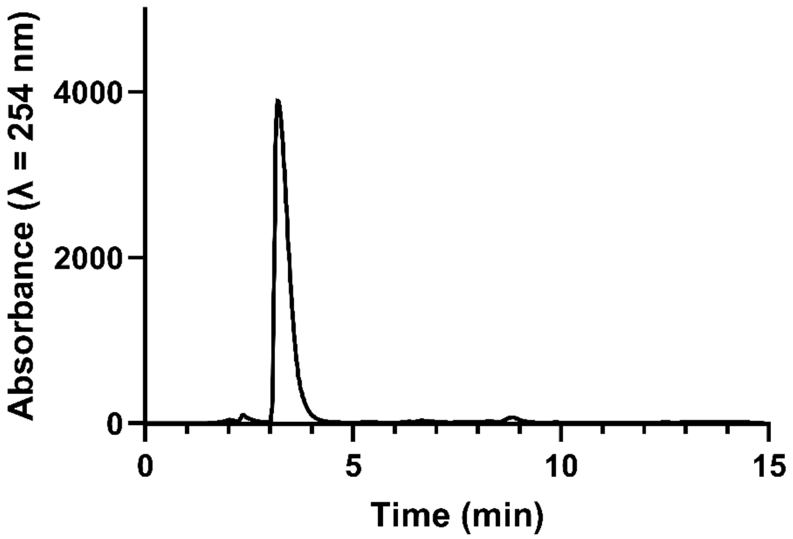

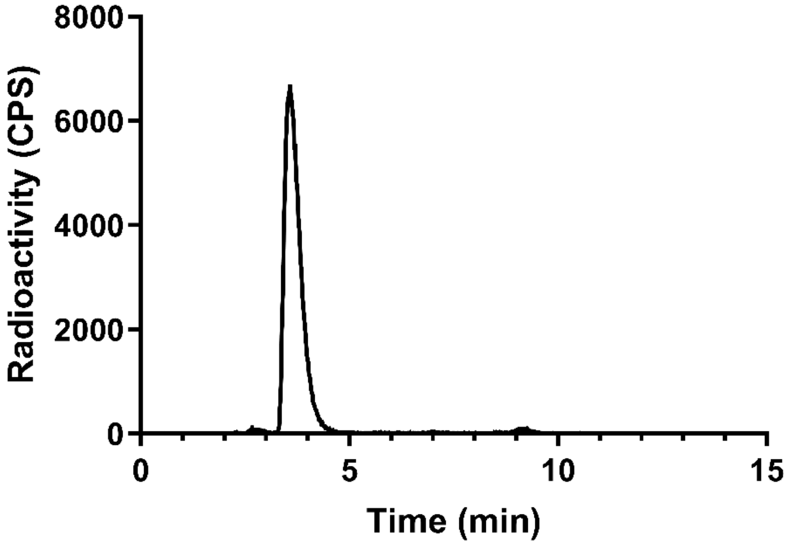

Figure S9. HPLC analysis of [^{195m}Pt]azoPt dissolved in PBS after an X-ray CT scan (Method 3). The presence of a single peak in both UV and radio-HPLC chromatograms confirms stability of the radiolabeled complex to X-ray irradiation.

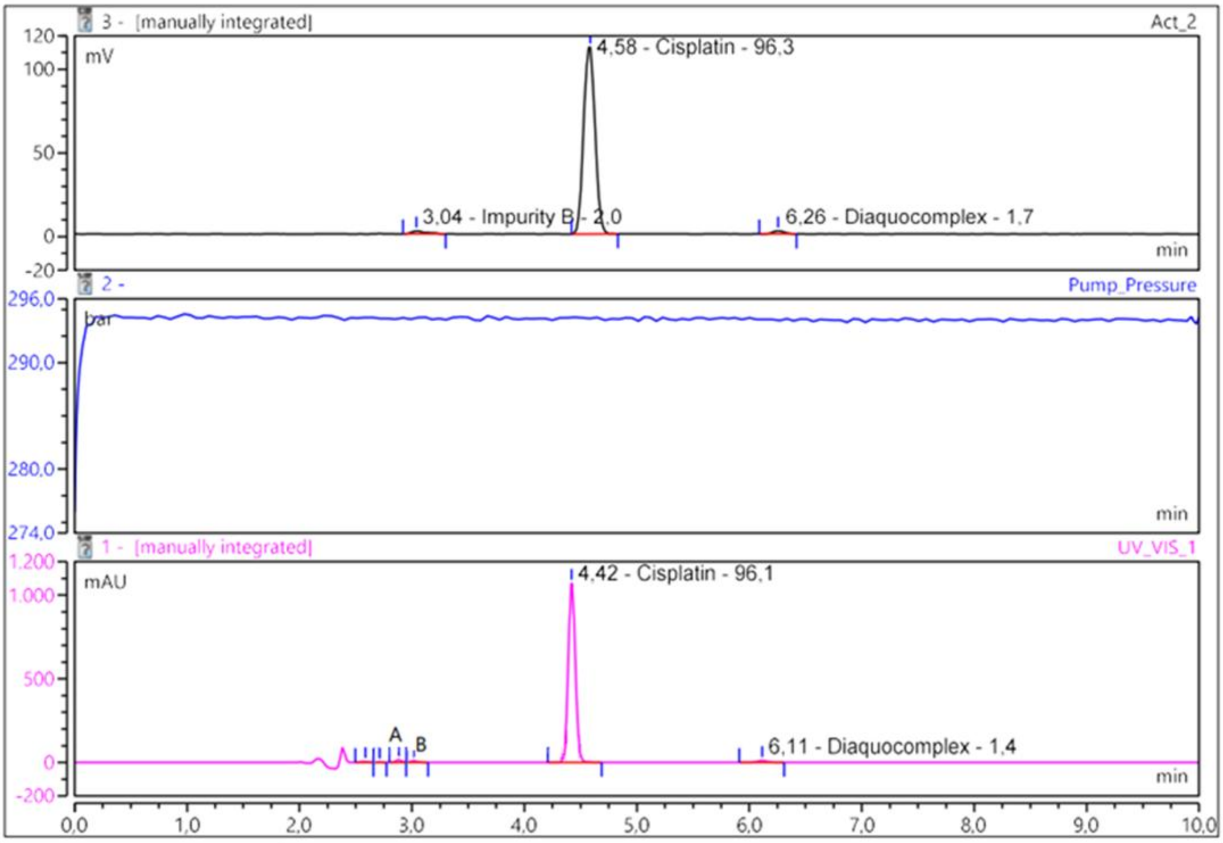

Peak No.	Ret.Time	Peak Name	Height	Area	Rel.Area	Amount
	min		mAU	mAU*min	%	mg/mL
1	2,59		4,83	0,34	0,4	n.a.
2	2,71		1,69	0,09	0,1	n.a.
3	2,88	Impurity A	17,70	0,97	1,2	n.a.
4	3,02	Impurity B	10,62	0,67	0,8	n.a.
5	4,42	Cisplatin	1070,83	77,84	96,1	0,942
6	6,11	Diaquocomplex	11,26	1,11	1,4	n.a.
Total:			1116,92	80,9993	100,0	

Figure S10. HPLC analysis of [^{195m}Pt]cisplatin confirming purity of the radiolabeled complex in both the radiochromatogram (top panel) and UV trace (bottom panel). Impurities A and B refer to transplatin and $[PtCl_3(H_2O)]^-$, respectively.

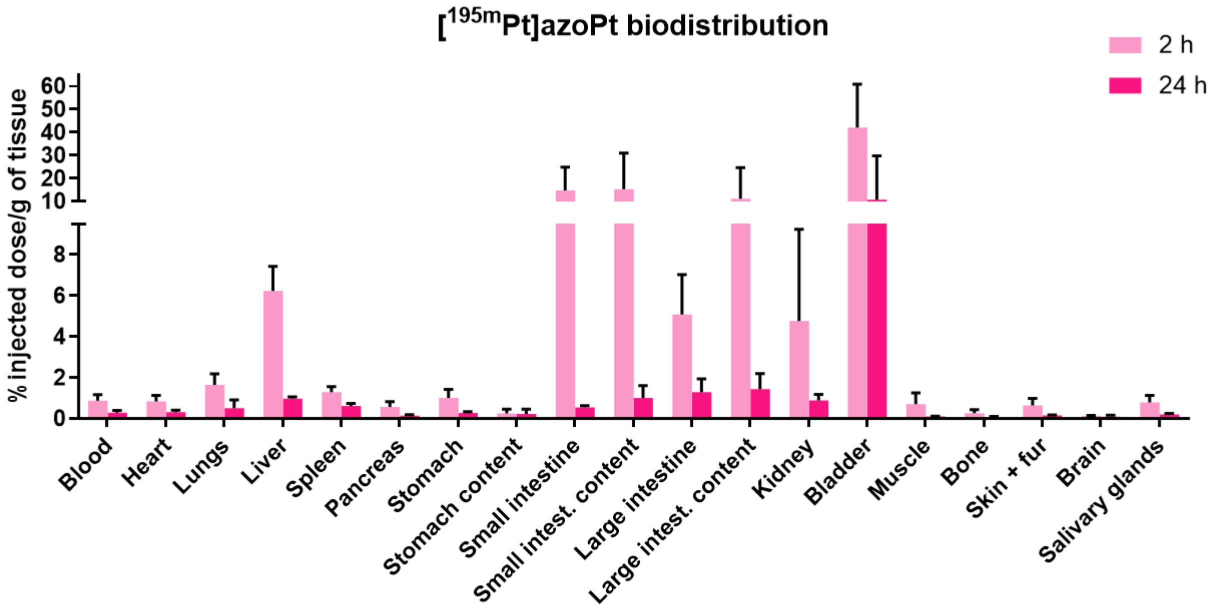

Figure S11. Biodistribution of [^{195m}Pt]azoPt in healthy mice, as determined by *ex vivo* organ gamma counting at 2 h and 24 h post-injection. Values are reported as mean ± standard deviation (n = 4).

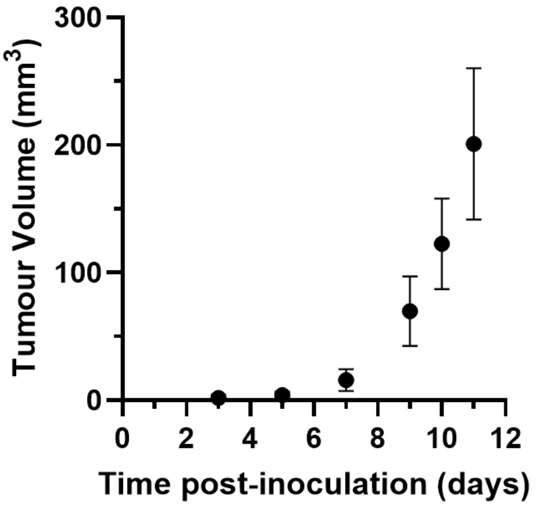

Figure S12. Tumor growth curve for BALB/c nude mice (7-9 weeks) inoculated with A2780 ovarian cancer cells (5 × 10^6 cells in 100 µL of PBS) on their right shoulder. Values are reported as mean ± standard deviation (n = 7).

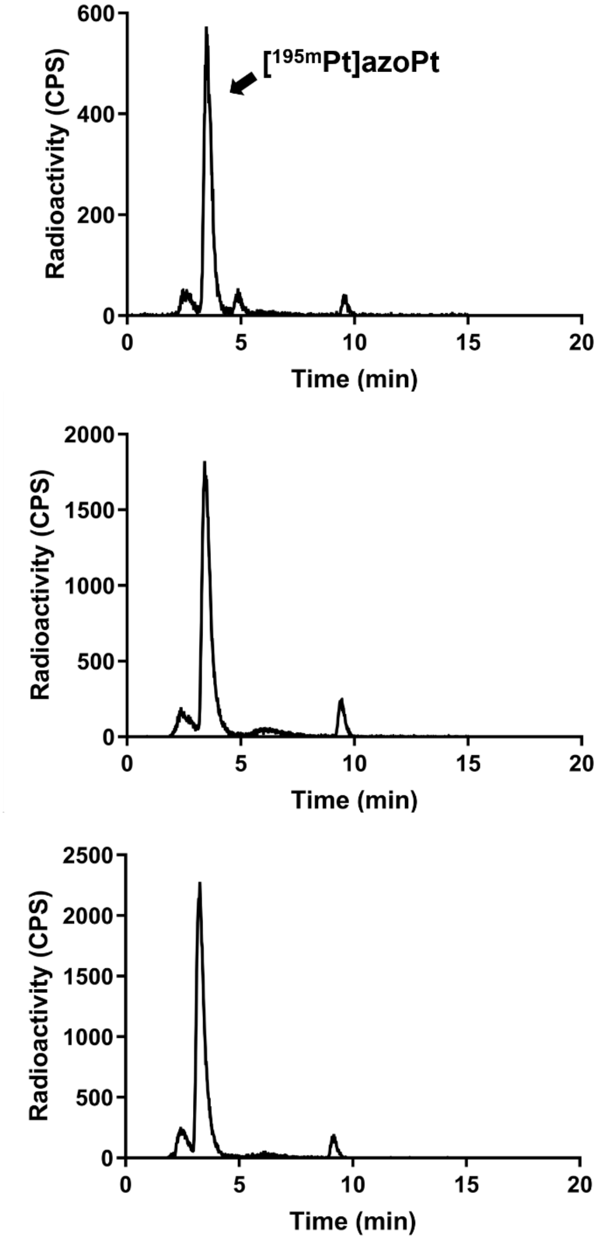

Figure S13. HPLC analysis of the urine collected from three different mice injected with [^{195m}Pt]azoPt (Method 3) at 2 h post-injection. Peaks with similar elution times are visible in all radio-chromatograms in slightly different proportions.

References

1. de Roest, R. H.; van Walsum, M. S.; van der Schilden, K.; Brakenhoff, R. H. *EJNMMI Res.* **2024**, *14*, 22
2. Hoeschele, J. D.; Butler, T. A.; Roberts, J. A.; Guyer, C. E. *Radiochim. Acta* **1982**, *31*, 27–36.
3. Hoogenkamp, D. S.; de Wit-van der Veen, B. J.; Hendriksen, J.; van der Schilden, K.; Nanne, J. A. M.; Belderbos, J. S. A.; Rossi, M. M.; Mooijer, M. P. J.; Funke, U.; Hendrikse, N. H.; Vogel, W. V.; Aalbersberg, E. A. *EJNMMI Res.* **2025**, *15*, 87.
4. Farrer, N. J.; Woods, J. A.; Salassa, L.; Zhao, Y.; Robinson, K. S.; Clarkson, G.; Mackay, F. S.; Sadler, P. J. *Angew. Chem., Int. Ed.* **2010**, *49*, 8905–8908.
